# Hallmarks of cancer and hallmarks of aging

**DOI:** 10.18632/aging.204082

**Published:** 2022-05-09

**Authors:** Mikhail V. Blagosklonny

**Affiliations:** 1 Roswell Park Comprehensive Cancer Center, Buffalo, NY 14263, USA

**Keywords:** oncology, carcinogenesis, geroscience, mTOR, rapamycin, hyperfunction theory

## Abstract

A thought-provoking article by Gems and de Magalhães suggests that canonic hallmarks of aging are superficial imitations of hallmarks of cancer. I took their work a step further and proposed hallmarks of aging based on a hierarchical principle and the hyperfunction theory.

To do this, I first reexamine the hallmarks of cancer proposed by Hanahan and Weinberg in 2000. Although six hallmarks of cancer are genuine, they are not hierarchically arranged, i.e., molecular, intra-cellular, cellular, tissue, organismal and extra-organismal. (For example, invasion and angiogenesis are manifestations of molecular alterations on the tissue level; metastasis on the organismal level, whereas cell immortality is observed outside the host).

The same hierarchical approach is applicable to aging. Unlike cancer, however, aging is not a molecular disease. The lowest level of its origin is normal intracellular signaling pathways such as mTOR that drive developmental growth and, later in life, become hyperfunctional, causing age-related diseases, whose sum is aging. The key hallmark of organismal aging, from worms to humans, are age-related diseases. In addition, hallmarks of aging can be arranged as a timeline, wherein initial hyperfunction is followed by dysfunction, organ damage and functional decline.

## Hallmarks of cancer: comparing apples and oranges

As depicted by Hanahan and Weinberg in 2000 [1], the circle schema of six hallmarks of cancer somewhat compares apples and oranges. https://els-jbs-prod-cdn.jbs.elsevierhealth.com/cms/attachment/428dbc2e-657c-429d-98f4-9910c7df1678/gr1_lrg.jpg.

The hallmarks themselves are exact, but they are not equal. For example, limitless replicative potential (cell immortality) cannot be directly compared to sustained angiogenesis. Cell immortality is revealed outside the host (extra-organismal level), for example, in cell culture where clonal cell lines can proliferate indefinitely without interaction with normal tissues. In contrast, sustained angiogenesis requires interaction of cancer cells with normal cells of several tissues. Angiogenesis can be only understood on the tissue level.

Second, cancer cells undergo Darwinian-type selection [2] for resistance to anti-growth signals, resistance to apoptosis and self-sufficiency in mitogenic signals. This trio represents three out of six hallmarks of cancer [1]. They can be combined in one super-hallmark: resistance to growth-limiting conditions [3]. (Note: The definition of oncogenic resistance to growth-limiting conditions was discussed previously [4]). Not only resistance to apoptosis and anti-growth signals but also self-sufficiency in mitogenic signals render cells resistant to growth-limiting conditions. Examples of growth-limiting conditions include lack of external mitogenic signals, cytostatic cytokines such as TGF-beta, cytotoxic carcinogens such as tobacco smoke, anti-cancer drugs, contact inhibition, glucose deprivation, cellular senescence, hypoxia, absence of nutrients and growth factors [5, 6]. For example, glucose deprivation selects for oncogenic Ras [6].

Whereas normal cells do not proliferate in growth-limiting conditions, cancer cells do. Resistance to growth-limiting conditions provides an immediate selective advantage. But what immediate advantages can be provided by cellular immortality? The cell cannot tell the future, that it will live in cell culture one day. Cellular immortality is selected indirectly as derived hallmarks [3], because the same mutations that provide resistance to growth-limiting conditions also make cells immortal, angiogenic, invasive and metastatic [1, 7, 8]. Cellular immortality, angiogenesis, invasion and metastasis are derived hallmarks.

Third, molecular alterations (e.g., DNA mutations) are absent in the six-hallmark circle by Hanahan and Weinberg [1]. As discussed by Gems and de Magalhães, the hallmarks do not include mutations (or genetic instability) because this hallmark is implicitly taken for granted [9]. In fact, Hanahan and Weinberg called it an enabling hallmark in their revised paper published in 2011 [7].

In 2005, I explicitly included the molecular hallmark (mutations) and suggested the hierarchical principle to arrange these hallmarks from molecular to organismal levels [5].

## Hierarchical model of hallmarks of cancer: arranging the oranges

**Figure 1:**
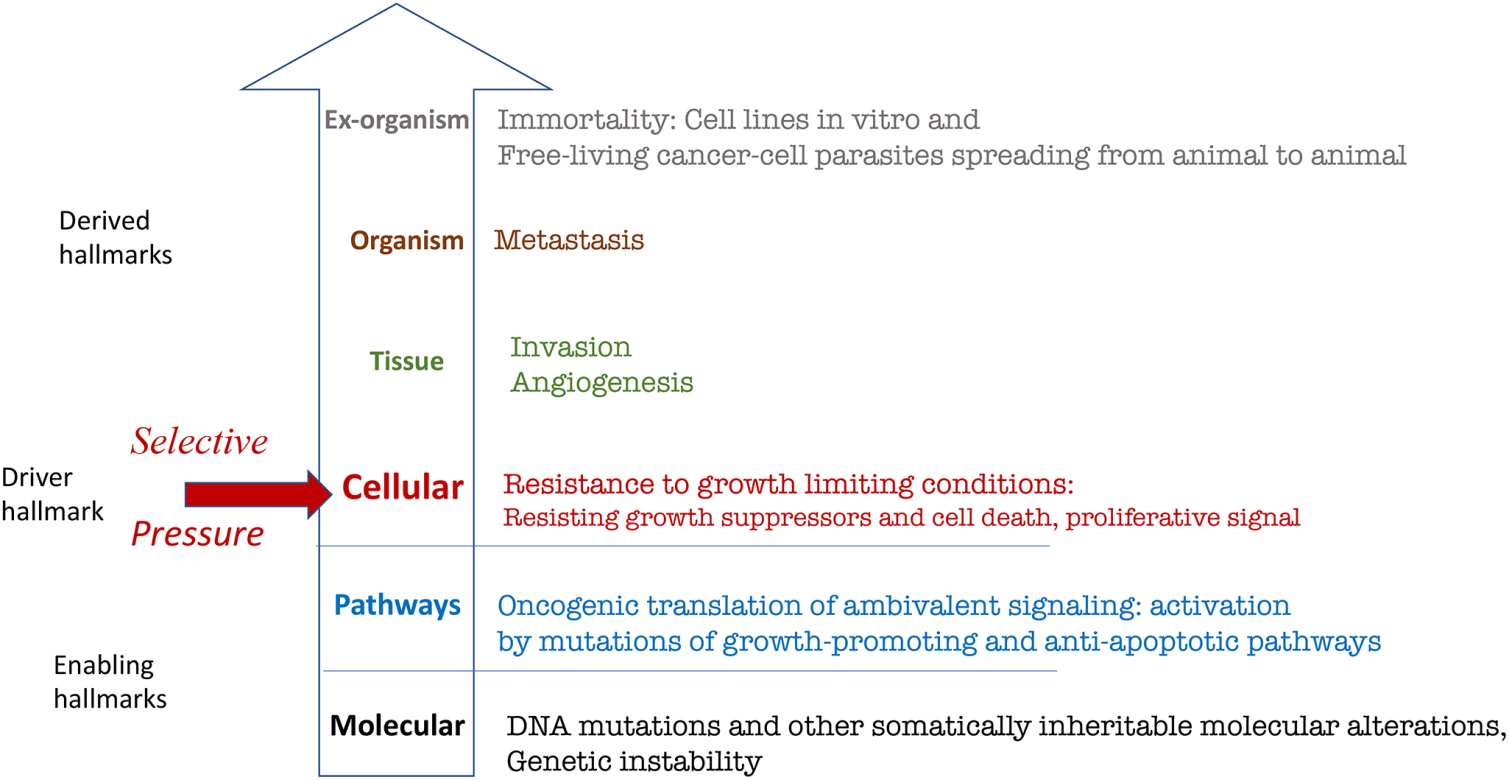
**Hierarchical representation (from molecular to organismal levels) of the original hallmarks of cancer based on Hanahan and Weinberg.** See text for explanation.

Here I present the hallmarks of cancer, depicted as a circle by Hanahan and Weinberg [1], not as the circle but hierarchically, from molecular levels to the organism (Figure 1).

### Molecular level: Somatically inheritable molecular alterations.

Genome instability is an enabling hallmark of cancer because it enables the acquisition of molecular alterations, such as DNA mutations, aneuploidy and epigenetic alterations [7]. Vogelstein et al. suggested that a typical human tumor contains relatively few driver gene mutations that each confers a growth advantage of 0.4% and numerous passenger gene mutations that confer no selective advantage [8, 10].

### Intracellular signaling pathways: Oncogenic translation of ambivalent signaling

Signal-transduction pathways are ambivalent, causing opposite outcomes depending on cellular context. Oncogenic mutations re-wire signal transduction pathways. For example, MAPK pathways can simultaneously induce cyclin D1 and CDK inhibitors, leading either to cellular proliferation or senescence [11]. Inactivation of CDK inhibitors such as p16 may translate this ambivalent signaling into proliferation [3, 12]. TGF-beta inhibits normal cell proliferation, but in cancer it can induce proliferation and invasion [7, 13].

Growth-promoting and mitogen/nutrient-sensing signaling pathways are constantly activated by mutations to promote growth and proliferation as well as self-sufficiency in mitogen signaling. This, in turn, is manifested as three hallmarks of cancer on the next hierarchical level: cellular. This trio can be combined as one super-hallmark of resistance to growth-limiting conditions.

### Cellular level: Resistance to growth-limiting conditions

Oncogenic mutations make cancer cells resistant to growth-limiting conditions (a definition of oncogenic-type of resistance was discussed previously [4]). This is the driver hallmark of cancer because it provides a selective advantage to cancer cells. Cells, capable of proliferation, are unicellular organisms in a Darwinian sense [2, 14, 15]. Selection can be “natural” (during carcinogenesis) and “artificial” (during cancer therapy) [14, 16]. For example, selection for therapy resistance increases oncogenic properties of cancer cells because many mutations in oncogenes and tumor suppressors that render cells drug-resistant also make them more oncogenic [5, 17–19]. Simultaneously, the same combination of mutations enables metastasis and other higher-level hallmarks. There is no direct selection for metastatic potential, angiogenesis and immortality. They are derived hallmarks.

### Tissue level: Invasion and angiogenesis

Cancer cells produce cytokines and enzymes, which enable the cells to invade and to attract normal cells of different tissues in order to sustain angiogenesis [7].

### Organismal level: Metastasis

Metastasis is the deadliest hallmark of cancer. Yet, there is no direct selection for metastatic potential. Direct selection for metastatic potential could take place only if metastases produced new metastases; in other words, if metastases reproduce. Simply, selection for cells resistant to growth-limiting conditions (survival and proliferation) brings about mutations that confer not only resistance, but also metastatic potential. There are no specific “metastasis” genes [8, 20]. They are the same oncogenes and tumor suppressors that act on cellular levels for the “trio” hallmark. Let us consider an analogy. If we select people for their ability to run faster, these selected people will also jump higher than average, although selection was not for jumping ability. The fastest runners are the farthest jumpers.

### Extra-organismal level: Cellular immortality

Some cancer cell lines live for more than half of a century in cell culture and for thousands of years in the wild. Originating in one animal, viable cancer cells are directly transmitted into unrelated hosts in a process similar to metastasis [21, 22]. Transmissible cancers have been observed in domestic dogs, the Tasmanian devil, hamsters and six bivalve species such as the soft-shell clam [23]. Canine transmissible venereal tumors (transmitted during sexual intercourse) may have originated thousands of years ago from the cells of a wolf or East Asian breed of dog [21–25]. The Tasmanian devil facial tumor disease [24] spreads through the Tasmanian devil population by transfer of cancer cells through biting [22]. [26]. Derived from a single original clam, leukemia-like cancer spreads among marine bivalves through sea water, leading to massive population loss [23, 27].

## Six levels rather than six hallmarks

The number of hallmarks of cancer is arbitrary. Some can be combined, and others can be added. Numerous authors have re-visited the hallmarks of cancer, adding hallmarks or suggesting a new set of hallmarks [28–37].

Some hallmarks of cancer may be pseudo-hallmarks. For example, visiting an oncologist is a “hallmark” of cancer. We can be 99% sure that if someone has 20 appointments in an oncology clinic, then this person has cancer. However, it would be ridiculous to include this pseudo-hallmark in Figure 1. And the hierarchical principle makes this impossible, because there is no level (among the six levels) to include it.

## Hallmarks of aging

**Figure 2:**
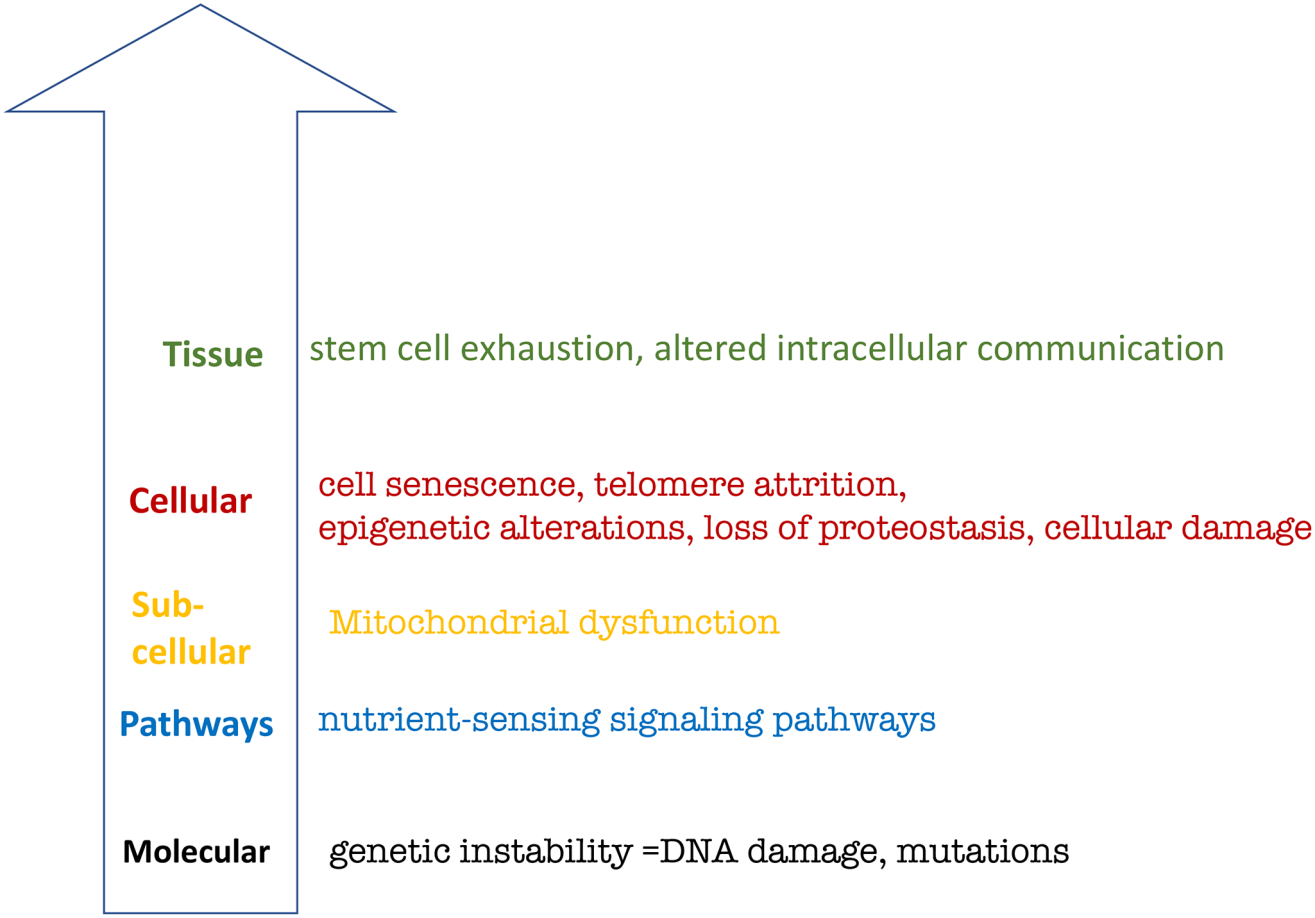
**Hierarchical representation of the hallmarks of aging based on López-Otín et al.** See text for explanation.

To start with, let us depict the hallmarks of aging suggested by López-Otín et al. [38] based on the hierarchical principle. This representation renders hallmarks tangible but reveals three shortcomings (Figure 2).

First is the lack of hallmarks on the organismal level. Yet, the main hallmark of organismal aging is age-related diseases in all species from *C. elegans* [39–42] to humans [39, 43]. Aging is the sum of all age-related diseases, which cause death “from aging”.

Second, the relationship between hallmarks on different levels are unclear.

Third, the inclusion of genetic instability as a hallmark is based on the theory that aging is caused by accumulation of molecular damage. The molecular damage theory was refuted by key experiments, as discussed in detail [44–51].

Yes, damage accumulates and is harmful and potentially lethal [52–55] but it is not life-limiting because aging caused by hyper-functional signaling terminates life first. The reason why mTOR-driven aging is life-limiting has been discussed [49, 56, 57].

It was also suggested that the levels of DNA repair needed to avoid cancer at a young age greatly exceeds the levels that are needed to prevent damage-induced aging during a normal lifetime [58]. As previously discussed, the role of molecular damage in cancer supports the role of mTOR-driven hyperfunction in aging [59].

**Figure 3:**
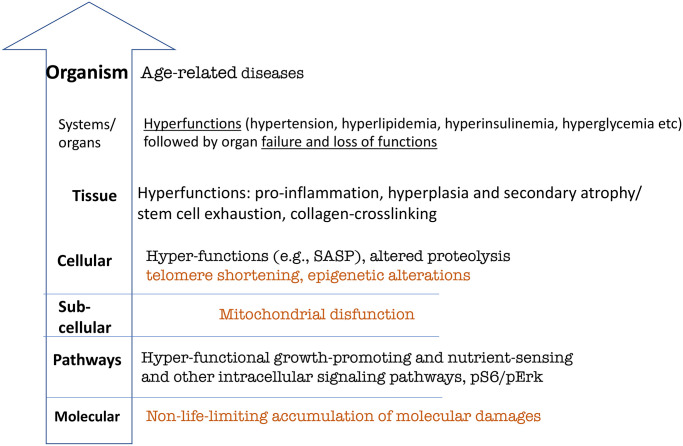
**Hierarchical hallmarks of aging based on hyperfunction theory, applicable to humans.** Non-life-limiting hallmarks are shown in brown color. See text for explanation.

Let us depict hallmarks of aging, according to the hyperfunction theory of aging (Figure 3).

## Hallmarks of aging and hyperfunction theory

The hyperfunction theory of aging was extensively reviewed previously [44, 45, 49, 56, 57, 60–66], and responses [60, 67] to its critics [68, 69] were also provided.

According to hyperfunction theory, aging is a continuation of developmental and reproductive programs that were not turned off upon their completion. Continuously active signaling pathways that initially drive developmental growth, drive aging later in life. Signaling pathways establish feedback loops, involving also gene expression and epigenetic modifications. These pathways become hyperfunctional, meaning that their activity is higher than optimal for longevity.

How does normal function become a deadly hyperfunction? Consider an analogy. When you pump air into an inflatable balloon, it grows in size. But when it reaches its intended size and you continue to pump air at the same rate, it will not grow further but instead will burst. This event can be compared with a stroke due to hypertension, resulting in brain damage. The brain is not damaged by life-long accumulation of molecular damage, but by hyperfunction, such as hypertension and hypercoagulation, thrombosis.

Hyper-function does not necessarily mean increased function. Even unchanged or slightly decreased activity of growth-promoting pathways, such as mTOR, can be hyperfunctional when developmental growth is completed. As an analogy, 55 mph on the highway is not speeding, but even 40 mph on the driveway is too fast.

Hyperfunction causes organ damage and functional decline. The accumulation of molecular damage is associated with decline, but it is hyperfunction that causes decline during a normal lifetime.

Unlike cancer, aging is not a molecular disease. Development is not driven by accumulation of molecular damage or mutations in signaling pathways, and aging is not either. Nutrient-sensing pathways (e.g., mTOR) are not altered by random mutations.

The lowest level of hallmarks of aging is a continuous activation of normal signal transduction pathways. Deactivation of these pathways by knockout of a single gene extends lifespan in animals [70–73]. Rapamycin, a drug that inhibits normal mTOR signaling, extends lifespan [74–77].

Hyperfunctional signaling directly drives age-related diseases. There are no longevity pathways/mechanisms inhibitable by pro-aging pathways such as mTOR. Pro-aging pathways do not drive aging by inhibiting longevity mechanisms. Why would nature create something that inhibits longevity mechanisms? Pro-aging pathways such as mTOR directly drive age- related diseases because they are a continuation of development.

## The key to understanding aging: life-limiting vs. non-life-limiting hallmarks

Among numerous harmful processes, only one can be life-limiting in a particular individual. If an animal dies from one cause, it cannot die from another cause even a day later. If quasi-programmed (e.g., mTOR-driven) aging is life-limiting, then accumulation of random damages cannot kill the organism.

López-Otín et al. [38] suggested three criteria for hallmarks of aging but two of them are criteria for both life-limiting and non-life-limiting processes: (1) hallmarks are observed during normal aging and (2) its experimental aggravation should decrease lifespan. However, experimental aggravation can make any process life-limiting. Telomere shortening becomes life- limiting in mice lacking telomerase, but their symptoms are drastically different from normal age-related diseases [78]. Although telomere shortening is associated with cardiovascular disease (CVD) in humans, patients with dyskeratosis congenita (DKC), a condition caused by short telomeres, do not die from CVD but from bone marrow failure (which is not a typical age-related disease) [79]. Hyperfunction theory explains how hyper-functional signaling leads to CVD in humans [80]. But telomere shortening cannot explain it.

Anything can shorten lifespan including starvation and the atomic bomb but they are not causes of aging. Only the third criterion matters: (3) its experimental amelioration should slow down aging and increase healthy lifespan. Not surprisingly, “the last criterion is the most difficult to achieve and not all of the hallmarks are fully supported yet by interventions,” as noted by López-Otín et al. [38]. In other words, they are not hallmarks of normal aging.

(Note: Even the third criterion is not sufficient to define a life-limiting hallmark.

Besides interventions may have off-target effects. For example, NAC, an antioxidant, is also a mTOR inhibitor [81]).

In conclusion, numerous deadly processes develop in parallel but only a few (or one) are life-limiting.

**Figure 4:**
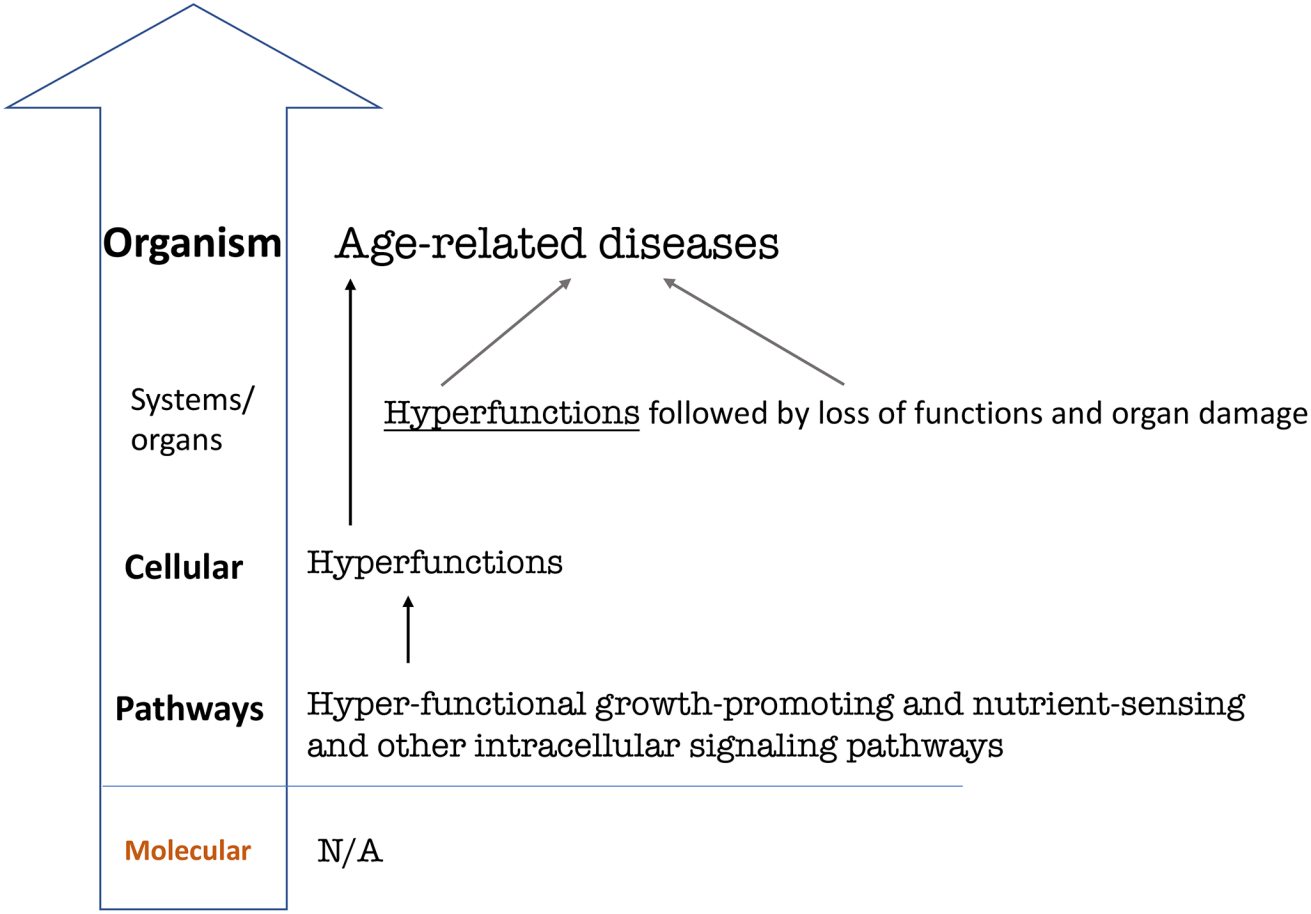
**Hierarchical hallmarks of aging based on hyperfunction theory, universal.** Hyperfunction of intracellular signaling pathways leads to cellular and systemic hyperfunctions, which in turn lead to age-related diseases on the organismal level [56]. Specific hyperfunctions and diseases may be different in different species and therefore are not shown. For example, human systemic hyperfunctions (e.g., hypertension, hyperlipidemia, hyperglycemia) and diseases (e.g., cardio-vascular diseases) differ from diseases in C elegans [40, 41].

Therefore, non-limiting hallmarks are not included in the version of life-limiting hallmarks of aging (Figure 4). This final re-presentation is generic and can be applied to any species, from *C. elegans* to humans.

## Aging as a selective force for cancer

Common cancers are age-related diseases. This cannot be explained by simple accumulation of mutations with age. For example, melanoma and lung cancer in smokers have atypically high mutation burden [8] but still develop at old age. Centenarians, who age slower, are protected from cancer. Rapamycin and calorie restriction slow aging in mice and prevent cancer.

As discussed, the selective force driving carcinogenesis is growth-limiting conditions, also named micro-environmental constraints in aging [16]. For example, the aging hematopoietic system selects for robust hematopoietic cells and such a preleukemic clone can originate leukemic clone [82]. Specifically, chronic inflammatory microenvironments in old age may select for cells harboring oncogenic mutations [83].

Chronic inflammation is a hyper-function and is in part mTOR-dependent [84–88]. An aging microenvironment puts stem cells on the path of hyper-activation [89] and geroconversion [90–92], leading to their exhaustion [89–92].

Mutations are necessary (with a few exceptions) but not sufficient for inducing cancer. The second requirement is selective force, favoring these mutations. Aging is a leading selective force.

One of the potential mechanisms of growth-limiting conditions that drive cancer progression is mTOR-dependent cellular senescence.

## Common hallmarks of cancer, aging and cell senescence

Cellular senescence is a two-step process: cell cycle arrest followed by geroconversion [93]. Like organismal aging, geroconversion is a continuation of growth driven in part by hyperfunctional mTOR. When the cell cycle is blocked by p21/p16, but growth-promoting pathways such as mTOR and MAPK are active, the cells become hypertrophic (large cell morphology) and hyperfunctional: beta-Gal staining (lysosomal hyperfunction) and SASP. A hallmark of cellular senescence is active mTOR pathway in non-proliferating cells. Rapamycin suppresses geroconversion to senescence [93–97]. Figuratively, organismal aging is a quasi-growth after developmental growth is completed.

In cancer, the PI3K/mTOR pathway is almost universally activated by mutations [98–100]. Figuratively, cancer cells are proliferating senescent cells. In organismal aging, cancer and cellular senescence, the same key signaling pathways, such as mTOR, are involved. This is why the same drugs, such as rapamycin, can suppress all of them.
